# Effect of a 1-year tailored exercise program according to cancer trajectories in patients with breast cancer: study protocol for a randomized controlled trial

**DOI:** 10.1186/s12885-023-10664-1

**Published:** 2023-03-02

**Authors:** Jiin Ryu, Eun-Young Lee, Jihee Min, Sujin Yeon, Ji-Won Lee, Sang Hui Chu, Hyangkyu Lee, Seung Il Kim, Jee Ye Kim, Seho Park, Justin Y. Jeon

**Affiliations:** 1 Department of Sport Industry Studies, Yonsei University, 50 Yonsei-ro, Seodaemun-gu, Seoul, 03722 Republic of Korea; 2 School of Kinesiology & Health Studies, Queen’s University, Kingston, ON Canada; 3 National Cancer Survivorship Center, National Cancer Center, Goyang, Republic of Korea; 4 Department of Family Medicine, Yonsei University College of Medicine, Seoul, Korea; 5 Mo-Im Kim Nursing Research Institute, College of Nursing, Yonsei University, Seoul, Korea; 6 Division of Breast Surgery, Department of Surgery, Yonsei University College of Medicine, 50 Yonsei-ro, Seodaemun-gu, Seoul, Republic of Korea; 7 Exercise Medicine Center for Diabetes and Cancer Patients, ICONS, Yonsei University, Seoul, Republic of Korea; 8 Cancer Prevention Center, Shinchon Severance Hospital, Yonsei Cancer Center, Yonsei University College of Medicine, Yonsei University, Seoul, Republic of Korea

**Keywords:** Breast cancer, Rehabilitation, Exercise

## Abstract

**Background:**

Patients with breast cancer undergo various treatments according to their tumor subtype and cancer stages within 1 year after being diagnosed. Each treatment may cause treatment-related symptoms that have negative impacts on patients’ health and quality of life (QoL) The symptoms can be mitigated when exercise interventions are appropriately applied to patients’ physical and mental conditions. Although many exercise programs were developed and implemented during this period, the effects of tailored exercise programs according to symptoms and cancer trajectories on patients’ long-term health outcomes have not yet been fully elucidated. Therefore, this randomized controlled trial (RCT) aims to investigate the effect of tailored home-based exercise programs on short-term and long-term physiological outcomes in patients with breast cancer.

**Methods:**

This 12-month RCT includes 96 patients with (stages 1–3) breast cancer randomly assigned to the exercise or control groups. Participants in the exercise group will receive an exercise program tailored to their phase of treatment, type of surgery, and physical function. During post-operative recovery, exercise interventions will be emphasized to improve shoulder range of motion (ROM) and strength. During chemoradiation therapy, exercise intervention will focus on improving physical function and preventing loss of muscle mass. Once chemoradiation therapy is completed, exercise intervention will focus on improving cardiopulmonary fitness and insulin resistance. All interventions will be home-based exercise programs supplemented with once-monthly exercise education and counseling sessions. The main outcome of the study is fasting insulin level at baseline, 6 months, and 1 year post-intervention. Our secondary outcomes include shoulder ROM and strength at 1 month and 3 months, body composition, inflammatory markers, microbiome, QoL, and physical activity levels at 1 month, 6 months, and 1 year post-intervention.

**Conclusion:**

This trial is the first tailored home-based exercise oncology trial to better understand the comprehensive phase-dependent short- and long-term effects of exercise on shoulder function, body composition, fasting insulin, biomarkers, and microbiome. The results of this study will inform the development of effective exercise programs tailored to the needs of patients with breast cancer post-operatively.

**Trial registration:**

The protocol for this study is registered with the Korean Clinical Trials Registry (KCT0007853).

**Supplementary Information:**

The online version contains supplementary material available at 10.1186/s12885-023-10664-1.

## Background

Globally, breast cancer is the most commonly diagnosed cancer (cancer incidence, 24.2%; and cancer mortality, 15.0%) among women [1], and 0.68 million women die from breast cancer in 2020 [1]. In Korea, the incidence of breast cancer has been increasing steadily, with 25,452 new cases, and it was the most common cancer among women in 2020 (24.7% of all cancer cases in women) [2]. Most patients with breast cancer undergo surgical resection and sampling or removal of axillary lymph nodes, followed by post-operative therapy [3]. Despite advances in surgical and therapeutic techniques, breast cancer surgery still results in various post-operative morbidities and complications.

The most frequently reported post-surgical complications are reduced shoulder range of motion (ROM), reduced shoulder strength, chronic pain, and sensory disturbances [4]. Patients also experience arthralgia, cellulitis, rotator cuff disease, shoulder impairment [5], axillary web syndrome [6], postural problems, or bothersome scarring [7] 1 month post-surgery. Lymphedema, the most invalidating post-surgical problem [8], can last for years after surgery. Furthermore, breast cancer surgery leads to greater psychological morbidity and poorer quality of life (QoL) in breast cancer survivors [6]. Although the impact of post-operative morbidities and complications on physical and psychological problems remains for a short time [9], upper limb dysfunction, such as decreased shoulder ROM and strength and pain tend to continue in the year after surgery [10]. In addition, gut microbiome has been shown to be associated with obesity [11], cellular dysplasia, and malignancies including breast cancer [12]. Dysbiosis could include disruption of bacterial homeostasis, which may influence breast tumorigenesis and potentially increase breast cancer risk [13]. Therefore, the clinical management of breast cancer requires a holistic multidisciplinary approach.

Approximately 1 month after surgery, most patients with breast cancer undergo chemoradiation therapy, which lasts for approximately 6 months. During this period, patients experience various treatment-related symptoms, such as nausea, vomiting, fatigue, and anorexia [9]. Since patients still do not fully recover from surgery, the additional burden of chemo-radiation treatment affects patients’ physical and psychological health. As a result, most patients with breast cancer experience side effects, such as loss of muscle mass, increased adiposity, and decreased physical fitness [14]. Furthermore, various breast cancer treatments have a negative impact on mental health, which can lead to mood problems, such as fear, anger, uncertainty about the future, depression, and anxiety [15–17]. Moreover, during the recovery period of 6–12 months after surgery, a variety of treatments for breast cancer using chemotherapy can induce metabolic changes, including decreased lean body mass and muscle strength, and a subsequent increase in body weight [18] and insulin resistance [19], which in turn may be associated with increased mortality among patients with breast cancer [17, 19].

Several studies have shown that exercise interventions can be an effective self-management strategy. Exercise has beneficial effects on fasting insulin levels [20], fatigue [21], body composition [22], and cardiovascular function [23] in patients with breast cancer [24, 25]. Furthermore, positive effects of exercise on the symptoms and prognosis of patients with breast cancer may have been mediated by changes in the microbiome [26]. Although many exercise programs have been developed and implemented, different types of breast cancer surgeries may cause damage to different muscles and fasciae, which would influence certain movements of the shoulder joints [27, 28]. Furthermore, each time point (1 month, 6 months, and 12 months) after surgery may require a different approach because the prevailing symptoms are likely to differ at different phases of post-operative recovery [28–30]. Furthermore, the ideal timing and types of exercise for patients with breast cancer experiencing various physical dysfunctions and symptoms have not yet been fully studied.

Therefore, the purpose of the current study is to evaluate the short- and long-term effects of a tailored exercise program according to the phase of treatment and the patient’s condition on fasting insulin levels, body composition, shoulder function, QOL, patient-reported outcomes, and the microbiome in patients with breast cancer for up to 12 months.

## Study objectives

### Objectives

#### Primary objective

- To evaluate the effect of the exercise program on changes in fasting insulin levels after exercise intervention (after chemotherapy, 12 months after surgery).

#### Secondary objective

- To evaluate the effect of the exercise program on changes in shoulder function, body composition, QOL, patient-reported outcomes, and the diversity and composition of the gut microbiome after an exercise intervention.

#### Exploratory objective

- To investigate changes in insulin levels, body composition, shoulder function, QOL, patient-reported outcomes, and the diversity and composition of the gut microbiome of patients with breast cancer from the day before surgery to 12 months after surgery.

## Methods

### Study design

A randomized, controlled, parallel-group pre-test and repeated post-test design will be applied to investigate the effect of the tailored exercise program for patients with breast cancer at each time point after surgery (post-operative day 1, third outpatient visit; 1 month from the baseline; during chemotherapy, fourth outpatient visit; 6 months from the baseline; during and after chemotherapy, and fifth outpatient visit; 12 months from the baseline; after chemotherapy). The exercise intervention and evaluation schedule can be changed based on the individual treatment period of chemotherapy (Table 1). The feasibility of the intervention and exploratory analysis of the effects of exercise on breast cancer patients were tested in a pilot study (KCT0006997). The results of the pilot study have been used to optimize the current study design by integrating videos for exercise sessions at home. This study will recruit participants from Severance Hospital, Yonsei University Health System, Seoul, Korea.

### Eligibility criteria

#### Inclusion criteria

Eligibility criteria will include the following: [1] women aged 19–70 years; [2] patients with histologically confirmed stage I, II, and III breast cancer; [3] medically suitable for evaluation and participation in exercise intervention; [4] no evidence of distant metastasis or locally recurrent breast cancer; and [5] ability to understand and provide written informed consent in the Korean language.

**Table 1:** Flow diagram for the schedule of enrollment, interventions, and assessments

TIME POINT	VISIT 1	VISIT 2 *[Phase 1]*	VISIT 3 *[Phase 1]*	VISIT 4 *[Phase 2]*	VISIT 5 *[Phase 3]*
	Baseline, (Before surgery)	POD 1	1 month from the baseline (± 2 weeks)	6 months from the baseline (± 1 month)	12 months from the baseline (± 1 month)
**ENROLLEMENT**					
Informed consent	○				
Sociodemographic information	○	○	○	○	○
**INTERVENTIONS**					
Exercise group		○	○	○	○
Usual care group					
**ASSESSMENTS**					
Body composition	○		○	○	○
Shoulder ROM & Strength	○		○	○	○
Physical activity	○		○	○	○
SPADI	○		○	○	○
Serum markers	○			○	○
Microbiome analysis	○			○	○
Nutritional status	○			○	○
QOL	○		○	○	○

Notes. POD, post-operative day; ROM: Range of motion; SPADI: Shoulder pain and disability index; QOL: Quality of life

#### Exclusion criteria

This study will exclude [1] patients who were scheduled for bilateral breast surgery; [2] immediate breast reconstruction surgery; [5] mental impairment leading to inability to cooperate; [6] current pregnancy or plans to become pregnant within the 1 year; and [7] any other condition or intercurrent illness (as assessed by the investigator).

#### Ethics and informed consent

The study protocol was approved by the Medical Ethics Committee of Severance Hospital, Yonsei University Health System, Seoul, Korea (4-2022-0027). This study will be conducted in accordance with the principles of the Declaration of Helsinki and Good Clinical Practice guidelines, including data and patient privacy protection. All the participants will provide written informed consent.

#### Sample size

The calculation for sample size was based on a previous study that evaluated the effects of 16 week mixed strength and endurance exercise on insulin levels in patients with breast cancer [31]. Based on a power of 80%, two-sided statistical significance level of 5%, and medium-sized effect size, the minimum required sample size would be 80 participants. Considering the dropout rate to be 20%, 96 patients with breast cancer are needed for the current study.

### Recruitment

**Fig. 1:**
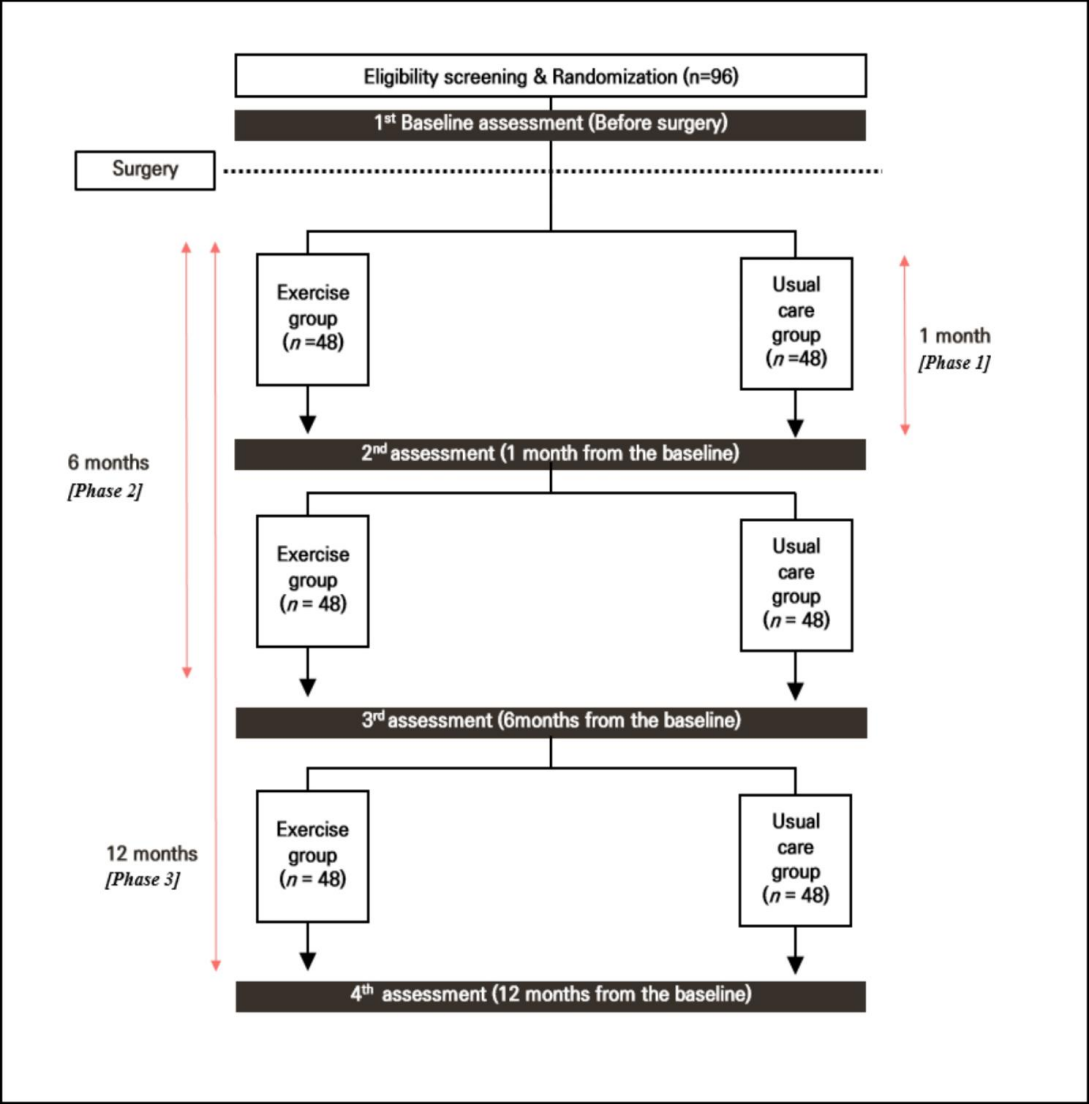
Overview of the protocol study

The current study will recruit participants for 12 months on a roll-in basis. Eligible participants will be recruited by physicians and specialists. Physicians will be informed about the study and exercise specialists will provide a study flyer that includes contact details and related information. Exercise specialists will obtain written consent from participants who meet the inclusion criteria. Eligible participants will undergo baseline medical examinations and physical performance tests. They will immediately receive appointments and necessary information about the study. Further details are presented in the study flowchart (Fig. 1).

## Randomization

Eligible participants will be randomly assigned to the exercise or control group, considering age, surgery method, and neoadjuvant chemotherapy as potential confounders. Each group would be matched into pairs such that the average age, surgical method, and neoadjuvant chemotherapy percentage are approximately equal (1:1 ratio). The exercise group will be provided with a systematic exercise program from post-operative day (POD) 1 to 12 months after surgery. The control group will receive the same preoperative and post-operative medical care and treatment as the exercise group (i.e., the usual care group thereafter), except for post-operative exercise. To evaluate the effects of the exercise intervention, measurements will be conducted five times for all the participants. The registration number of the Korean Clinical Trials Registry is KCT0007853. Data will be de-identified for analysis, and only study leaders at the university hospital will have access to identifiable information for all participants.

### Intervention

#### Usual care group

Participants in the usual care group will be instructed to continue their routine activities. Twelve months after surgery, the usual care group will receive exercise counseling, exercise education, and an exercise diary, based on the measurement results.

#### Exercise group

**Fig. 2:**
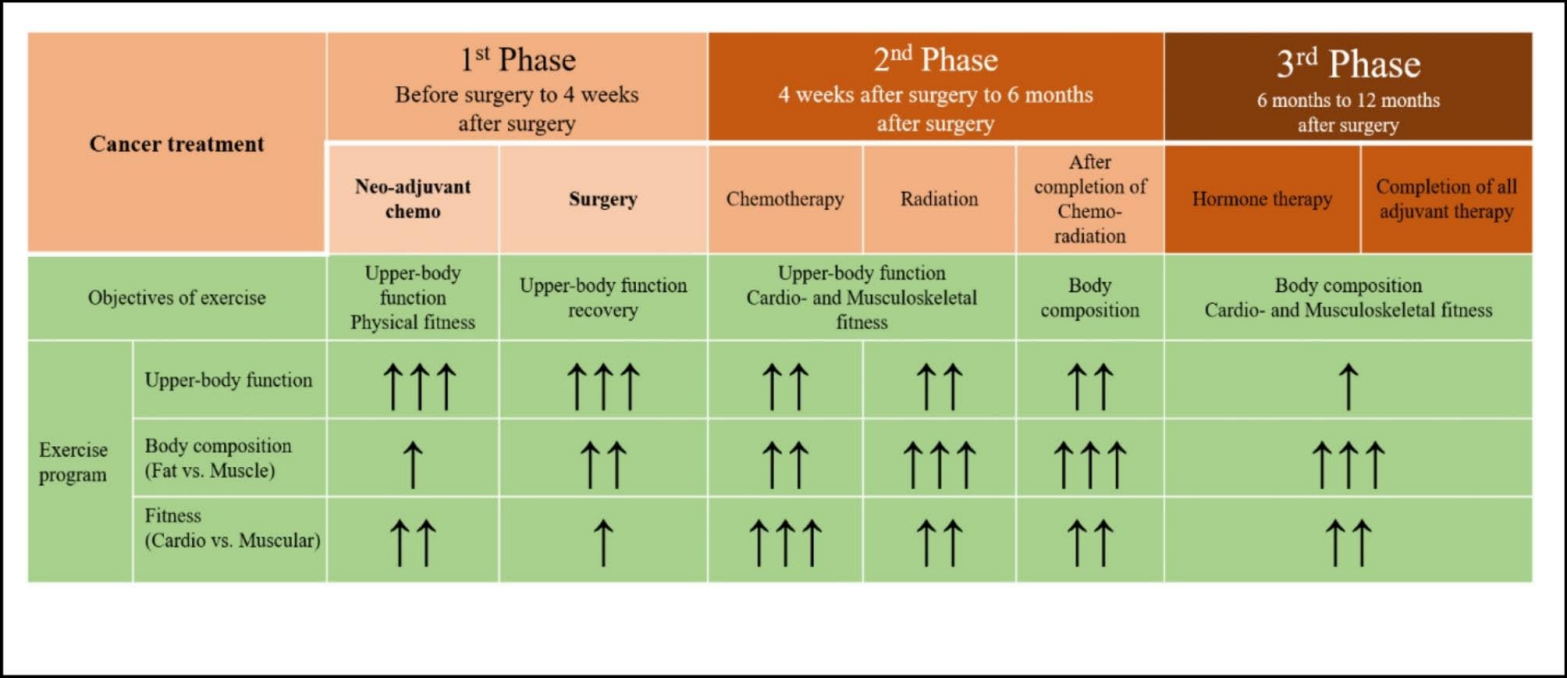
Overview of the Three Phases

The exercise group will receive the usual care and exercise programs based on previously used exercise programs in clinical trials (KCT0006997). The exercise program according to the phases of cancer trajectory will consist of supervised and home-based exercises. Up to 1 month after surgery, weekly supervised exercises and daily home-based exercises will be provided. Thereafter, supervised exercise will be provided once per month, along with home-based exercise. The purpose of the supervised exercise session was to educate participants, enabling them to perform exercise daily at home with the help of exercise videos that included detailed instructions on how to perform each exercise. The exercise programs will be tailored based on the patient’s condition and each time point after surgery [1 month from the baseline (± 2 weeks); Phase 1, 6 months from the baseline (± 1 month); Phase 2, 12 months from baseline (± 1 month); Phase 3] (Fig. 2).

#### Exercise program

Figure 2. Overview of three phases.

The exercise program was developed (Steps 1 to 9) based on a review of the literature [32, 33], an established understanding of patient characteristics [28, 34], expert panel meetings, feasibility studies,

and revision of the exercise program [35, 36]. The exercise program was divided into three phases according to the individual treatment period: surgery, chemoradiation therapy, hormone therapy, and the patient’s condition (Fig. 2). The exercise program consists of seven different intensities, standards for the application of exercise programs (Supplementary 1,2,3,4,5,6,7), and an exercise diary. Post-operative breast cancer patients and medical staff reviewed the safety of the exercises and the feasibility of the exercise evaluation design through a feasibility study [35]. The program included stretching and resistance exercise components for recovery of shoulder ROM, recovery of shoulder strength, reduced pain and discomfort after breast cancer surgery, and stretching of tight muscles (e.g., pectoralis major and minor muscles), and resistance exercises for muscle weakness (e.g., trapezius and rhomboid major and minor muscles).

#### *Phase* 1 [Baseline ~ 1 month from the baseline (± 2 weeks)]

The goal of the exercise program during the first phase is to facilitate the recovery of shoulder ROM and strength in breast cancer patients post-operatively. Prescription of exercise is emphasized to improve shoulder recovery and maintain whole-body function. Therefore, exercise programs should include tailored calisthenics exercises based on the patients’ shoulder joint condition. During this phase, no structured aerobic exercise program will be provided to patients. The very low-intensity calisthenics exercise program consists of nine different exercises. Specific exercises include neck stretching, shoulder rotation stretching, shoulder retraction, a clasp with a small ball, pendulum exercise, pelvic tilt on the wall, back extension, calf raise, and seated leg extension on a chair. For each exercise, the participants will perform 1–5 sets of 3–5 repetitions or 3–5 s with an accepted form recommended by exercise specialists (Supplementary 1). The very low-to low-intensity calisthenics exercise program consists of 11 different exercises. The specific exercises include neck stretching, shoulder rotation stretching, shoulder retraction, a clasp with a small ball, pec-dec fly, pendulum exercise, child pose, back extension, pelvic tilt on the wall, calf raise, and seated with the leg extended on a chair. For each exercise, participants will perform 1–5 sets of 3–5 repetitions or 3–5 s with an accepted form recommended by exercise specialists (Supplementary 2). The low-intensity calisthenics exercise program consists of 11 exercises. The specific exercises include neck stretching, shoulder rotation stretching, shoulder retraction, a clasp with a small ball, pec-dec fly, pendulum exercise, child pose, back extension, pelvic tilt on the wall, calf raise, and seated leg extension on a chair. For each exercise, participants will perform 1–5 sets of five repetitions or 5 s with an accepted form recommended by exercise specialists (Supplementary 3).

#### *Phase* 2 [POD 1 month ~ 6 months from the baseline (± 1 month)]

The goal of the exercise program during this phase is to obtain full shoulder ROM and strength recovery and to maintain cardiopulmonary fitness and muscle mass. Tailored calisthenics and aerobic exercises will be implemented. During this period, participants will be encouraged to engage in at least 150 min of moderate-intensity exercise in conjunction with detailed daily calisthenics exercise weekly. The low-to moderate-intensity calisthenics exercise is composed of 11 different exercises with low to moderate difficulties. The specific exercises include neck stretching, shoulder rotation stretching, shoulder retraction, a clasp with a small ball, pec-dec fly, pendulum exercise, child pose, back extension, pelvic tilt on the wall, calf raise, and sitting with the leg extended on a chair. For each exercise, participants will perform 1–5 sets of five repetitions or 5–10 s with an accepted form recommended by exercise specialists (Supplementary 4). The moderate-intensity calisthenics exercise program consists of 10 different exercises. The specific exercises include the following: neck stretching, shoulder rotation stretching, cross-chest stretching, scapular retraction, bird dog, child pose, back extension, pelvic tilt on the wall, chest stretching, and chair squats. For each exercise, participants will perform 1–10 sets of five repetitions or 5–10 s with an accepted form recommended by exercise specialists (Supplementary 5). For participants whose shoulder ROM or strength have not fully recovered, tailored shoulder exercises will be included.

#### *Phase* 3 [6 months (± 1 month) ~ 12 months from the baseline (± 1 month)]

The goal of the exercise program during this phase is to improve cardiopulmonary fitness and increase muscle strength and mass. Tailored calisthenics and aerobic exercises will be implemented. During this period, participants will be encouraged to engage in at least 150 min of moderate-to high-intensity physical activities (at least 75 min of high-intensity physical activity will be recommended) in conjunction with detailed daily calisthenics exercise per week. The moderate-to-vigorous-intensity calisthenics exercise is composed of 11 different exercises. Specific exercises include neck stretching, modified chest stretching, cross-chest stretching, scapular retraction, chair squat, child pose, back extension, bird dog, pelvic tilt on the wall, chest stretching, and Y stretching. For each exercise, participants will perform 1–10 sets of five repetitions or 5–10 s with an accepted form recommended by exercise specialists (Supplementary 6). The vigorous-intensity calisthenics exercise program consisted of 12 exercises. The specific exercises include neck stretching, modified chest stretching, modified pec-dec fly, cross-chest stretching, bicycle crunch, bird dog, wall push-up, chest stretching, Y-stretching, and dynamic squat. For each exercise, participants will perform 1–10 sets of five repetitions or 5–10 s with an accepted form recommended by exercise specialists (Supplementary 7).

### Study outcome measures

#### Primary outcome measures

[The evaluation schedule: at baseline, 6 months from the baseline (± 1 month), and 12 months (± 1 month; Primary outcome)]. After an overnight fast (≥ 10 h) blood would be obtained from the participants, and separated serum samples will be immediately stored at − 80 °C until they are assayed. Fasting insulin levels will also be measured using chemiluminescent enzyme immunoassays (Roche, IN, USA).

### Secondary and ancillary Outcomes Measures

#### Shoulder ROM

[The evaluation schedule: at baseline, 1 month from the baselines (± 2 weeks), 6 months from the baseline (± 1 month), and 12 months (± 1 month)]. Shoulder passive ROM, including flexion, abduction, and extension, will be measured using a goniometer (goniometer bending iron 29-5900, Pakistan) and standardized assessments (Norkin, 2004). All measurements will be performed on both the affected and unaffected arms, and the mean values will be used.

#### Shoulder strength

[The evaluation schedule: at baseline, 1 month from the baselines (± 2 weeks), 6 months from the baseline (± 1 month), and 12 months (± 1 month)]. Shoulder strength will be measured using a handheld dynamometer (j-tech Medical Industries Inc., UT, USA) in pounds (lb). The strength of peak muscle force will be measured using maximal voluntary isometric contraction (MVIC) in flexion, abduction, and extension [37]. All muscular strength measurements will be performed twice with both the affected and unaffected arms, and the average values will be used for the analysis.

### QOL (QOL)

[The evaluation schedule: at baseline, 1 month from the baselines (± 2 weeks), 6 months from the baseline (± 1 month), and 12 months (± 1 month)]. QOL will be measured using the EuroQOL-5 dimensions-5 levels (EQ-5D-5 L) descriptive system. Health-related QOL is divided into five dimensions (mobility, self-care, usual activities, pain/discomfort, and anxiety/depression), and there are five levels of response for each question (no problems, slight problems, moderate problems, severe problems, and extreme problems). Additionally, the EQ-5D-5 L assesses overall health status using a visual analog scale (EQ-VAS), a 100-mm scale with a score ranging from 0 (the worst health you can imagine) to 100 (the best health you can imagine). The Korean version of EQ-5D-5 L that will be used in this study was validated in a previous study [38].

#### Shoulder pain and disability index (SPADI)

[The evaluation schedule: at baseline, 1 month from the baselines (± 2 weeks), 6 months from the baseline (± 1 month), and 12 months (± 1 month)]. The shoulder pain and disability index (SPADI) is a self-administered questionnaire for shoulder pain with a total of 13 questions (five questions assessing shoulder pain and eight questions assessing disability). The reliability and validity of the Korean SPADI have been verified in a previous study (Seo, Lee, Jung, & Chung, 2012). Pain-free or most comfortable state scores 0, and painful or most uncomfortable state scores 10 for each SPADI item. Response scores within each subscale are summed, and a score out of 100 is given.

#### Anthropometric parameters and physical activity (PA)

[The evaluation schedule: at baseline, 1 month from the baselines (± 2 weeks), 6 months from the baseline (± 1 month), and 12 months (± 1 month)]. Body composition and Body mass index will be measured using InBody (BIO-SPACE®, InBody 720, Korea). The PA of participants will be measured using the Global Physical Activity Questionnaire (GPAQ), developed by the World Health Organization (WHO). It asks for any PA (including exercise) performed for at least 10 min during a typical week. Respondents will also report the time spent in PA performed at the workplace, transport, recreation, and sedentary behavior. The reliability and validity of the Korean GPAQ were examined in a previous study [39]. We will calculate PA as metabolic equivalent (METs) based on the GPAQ. Surgical complications that occurred within 30 days after surgery will be monitored through chart review. Surgical complications include surgical infection, wound complications (e.g., hematoma, wound dehiscence), seroma, and reoperation after breast cancer surgery.

#### Serum markers

[The evaluation schedule: at baseline, 6 months from the baseline (± 1 month), and 12 months (± 1 month)]. Insulin resistance will be assessed using the HOMA-IR index [fasting insulin (µIU/mL) × fasting glucose (mmol/L)/405]. The serum tumor markers CEA and CA 15 − 3 will be measured using chemiluminescent enzyme immunoassays (Roche, IN, USA). TNF-α levels will be measured using a commercially available enzyme-linked immunosorbent assay (R&D, Minneapolis, MN, USA). High-sensitivity C-reactive protein (hs-CRP) levels will be measured using a latex-enhanced immunoturbidimetric assay using an ADVIA 1650 chemistry system (Bater). Adiponectin level will be measured using an enzyme immunoassay kit (Mesdia, Seoul, Korea). Serum levels of fasting glucose, total cholesterol (TC), triglycerides (TG), and high-density lipoprotein cholesterol (HDL-C) will be measured from serum using an ADVIA 1650 chemistry system (Siemens, NY, USA).

#### Nutritional status and Microbiome analysis

[The evaluation schedule: at baseline, 6 months from the baseline (± 1 month), and 12 months (± 1 month)]. Several studies have suggested that the gut microbiota is mainly driven by nutritional status [40–42]. Participants’ nutritional status will be measured using a food frequency questionnaire (FFQ). The FFQ was based on food records from the Korea Health and Nutrition Examination Survey (KHANE) in 1998 and consisted of 109-food based questions and 29-dish based questions [43]. The FFQ has a reasonable relative validity for nutrient intake in Korean adults [43]. Stool samples will be collected by patients and the samples will be stored at − 80 °C until analysis. The total DNA will be extracted directly from the fecal samples using the FastDNA SPIN Kit for Soil (MP Biomedicals, Santa Ana, CA, USA), following the manufacturer’s instructions. Polymerase chain reaction (PCR) will be used to amplify the V4 region of the 16 S rRNA gene, which will be analyzed using Illumina MiSeq DNA sequencing. The data will be analyzed using the EzBioCloud system (www.ezbiocloud.net). Microbiome analyses, including quality control, OUT picking, sequence databases, and sequencing primers, will be performed according to the protocol described by Yoon et al. [44].

#### Statistical analysis methods

All analyses will be performed using the SPSS version 26.0 software (IBM Corp., Armonk, NY, USA). A Chi-square test for categorical variables and an independent t-test for continuous variables will be used to test group differences at baseline. Two-way repeated-measures ANOVAs will be conducted to observe the effects of the intervention on fasting insulin levels, shoulder ROM and function, body composition, inflammatory markers, microbiome, QOL, and physical activity levels. Paired t-tests will be performed to examine the difference between outcome measurements and baseline values, while independent t-tests will be employed to examine group differences in variables at each time point. The Bonferroni correction method will be used to correct for multiple tests. Analysis of covariance (ANCOVA) will be applied to test the effect of an exercise intervention on body composition, PA, and complications, since these variables will be measured after adjusting for baseline values. First, from baseline to 1 month from the baseline will be calculated and then the differences between groups will be examined after adjusting for baseline values and surgical methods. The same methods will be used for 6 and 12 months.

## Results

### Discussion

Most patients with breast cancer undergo intense cancer treatments, including surgery, chemotherapy, and hormone therapy, depending on the stage and subtypes of cancer. These treatments result in shoulder dysfunction [28], cardiotoxicity [45], fatigue [46], sarcopenia, and insulin resistance [47], which are known determinants of poor QoL and prognosis [48]. Supervised exercise has been identified as a viable and supplemental approach for the rehabilitation of patients with breast cancer [49]. Numerous studies have shown that exercise has a significant clinical benefit in patients with breast cancer and have recommended engaging in regular exercise. Exercise is an alternative approach for mitigating the negative effects of cancer treatment and functional limitations [50, 51]. However, most previous studies have investigated the benefits of exercise on the above-mentioned outcomes in patients with breast cancer before, during, or after adjuvant chemoradiation therapy as separate studies in different samples. Moreover, there is a lack of information on exercise programs for patients with cancer during and following treatment periods for health maintenance. It is logical to think that tailored exercises should be applied to breast cancer patients because most breast cancer patients experience different treatment side effects after surgery. Therefore, we aimed to test the efficacy of a tailored exercise program, based on three different recovery phases (i.e., Phase I [surgery to 1 month], Phase II [1 month to 6 months], and Phase III [7 months to 12 months]), in the same research participants on insulin resistance as well as other symptom-related health outcomes.

During Phase I (surgery to 1 month), the most prevalent comorbidity following breast cancer surgery is poor shoulder function, including a decrease in ROM and impaired strength [52]. One recent study showed that recovery of ROM differs by surgery type; however, shoulder strength was reduced regardless of the surgery type [28]. It was also reported that reduced shoulder strength in the unaffected arm suggests reduced shoulder strength in both the affected and unaffected arms due to lack of utilization [28]. A recent study found that shoulder ROM and strength recover to pre-surgery levels within 1 month after surgery if a proper exercise program is implemented [35]. Taken together, these studies suggest that exercises focused on the recovery of shoulder function should be emphasized during this phase.

During the chemotherapy period (phase 2), patients with breast cancer typically experience muscle mass loss [53], insomnia [54], cardiotoxicities [45], and fatigue [46]. Exercise intervention improves these parameters [55] and the recent American Society of Clinical Oncology guidelines clearly recommend oncology providers recommend aerobic and resistance exercises during active treatment with curative intent to mitigate the side effects of cancer treatment [51]. Furthermore, exercise intervention during chemotherapy may improve the chemotherapy completion rate [56] and reduce the hospitalization rate [57], although the effect of exercise on chemotherapy completion remains controversial [58]. Furthermore, during hormone therapy, more than 60% of patients experience joint and muscle pain [59], which can be improved through proper exercise [60]. Although exercise could be beneficial for breast cancer survivors during this period, shoulder dysfunction, fatigue, and low fitness levels could be barriers to exercise. Therefore, exercise programs during this period should be tailored to the patients’ physical condition with the aim of maintaining muscle mass and fitness.

During the first year after cancer surgery (Phase III), most patients with breast cancer experience an increase in fat mass and decreased muscle mass, insulin resistance, and the risk of recurrence and mortality [61, 62]. In addition, fasting C-peptide levels in patients with breast cancer are associated with a 35% increased risk of death due to breast cancer [63]. Exercise is an effective self-management tool associated with body composition and reduced fasting insulin levels among breast cancer survivors [31, 33, 64, 65]. Thus, the effects of exercise on breast cancer outcomes may be mediated by insulin resistance and inflammatory markers. The intervention duration in most exercise intervention studies for cancer survivors was less than 6 months, and these studies implemented exercise after the completion of adjuvant therapies. In this regard, the exercise program developed for this trial tailored the exercise program to patients’ physical conditions, and cancer treatment-related symptoms were unique. Thus, our results will add new knowledge to existing knowledge.

Our study adds new knowledge in several aspects. First, our tailored exercise intervention will improve insulin resistance and would confirm the factors responsible for such improvement. Second, this trial will measure the microbiome of patients with breast cancer, which may be correlated with prognosis [66–68], along with other biomarkers. Therefore, the outcome of this trial will provide a clear explanation for changes in other physiological and psychological outcomes via changes in the microbiome. In addition, this randomized study will show that exercise programs according to phases of cancer trajectory have important clinical implications. It might influence patients’ lifestyle modification by changing their perceptions; hence, cost-effectiveness outcomes of patients’ lifestyle modification could potentially be expected.

## Conclusion

In summary, this trial is the first long-term tailored home-based exercise oncology trial to understand the long-term effects of exercise on fasting insulin levels, a surrogate indicator of cancer as well as an important indicator of metabolic health. The results of this trial highlight the importance of developing tailored exercise programs according to the phases of the cancer trajectory for patients with breast cancer, which will ultimately help with their recovery. These findings related to the program according to phases of cancer trajectory may represent a method of reducing breast cancer morbidity and mortality by increasing exercise and other positive effects. Therefore, tailored home-based exercises could have positive effects on a patient’s general condition.

## Electronic supplementary material

Below is the link to the electronic supplementary material.

Supplementary Material 1

## Data Availability

Not applicable.

## Abbreviations

ANCOVA: Analysis of covariance
GPAQ: Global Physical Activity Questionnaire
hs-CRP: High-sensitivity C-reactive protein
MVIC: Maximal voluntary isometric contraction
PA: Physical activity
POD: Post-operative day
QOL: Quality of life
RCT: Randomized controlled trial
ROM: Range of motion
SPADI: Shoulder pain and disability index
