# Supplementary material for: Effect of a 1-year tailored exercise program according to cancer trajectories in patients with breast cancer: study protocol for a randomized controlled trial
Source: BMC Cancer. 2023 Mar 2;23:200. doi: 10.1186/s12885-023-10664-1 (PMC9983270; doi:10.1186/s12885-023-10664-1)
Supplement: Supplementary file 1 — Supplementary Material 1 [file 12885_2023_10664_MOESM1_ESM.docx]

| **Supplementary 1.** Daily exercise program of the study (very low intensity; *Phase 1*) |
| --- |

| **No** | **Content** | **Psition** | **Frequency** | **Time** | **Type** | **QR code** |
| --- | --- | --- | --- | --- | --- | --- |
| 1 | Neck stretching | Standing  &  Chair | At least 1/  Back and forth, side to side | 5 s | Stretching | 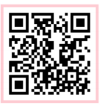 |
| 2 | Shoulder  rotation  stretching |  | At least 5/  Back and forth | 5 reps | Stretching |  |
| 3 | Row or  Shoulder  retraction |  | At least 5/  Back and forth | 5 s | Stretching |  |
| 4 | Clasp with  a small ball |  | At least 5 /  Right hand and left hand | 5 s | Resistance,  Isometric |  |
| 5 | Pendulum  exercise |  | At least 10/  surgery arm (only) | 5 rep | Stretching |  |
| 6 | Pelvic tilt  on wall |  | At least 5 | 5 s | Resistance,  Isometric |  |
| 7 | Back extension  exercises |  | At least 1 | 5 s | Stretching |  |
| 8 | Calf raise  exercise |  | At least 5 | 3 s | Resistance |  |
| 9 | Seated leg extension on chair |  | At least 5 | 5 s | Resistance |  |

| **Supplementary 2.** Daily exercise program of the study (very low to low intensity; *Phase 1*) |
| --- |

| **No** | **Content** | **Psition** | **Frequency** | **Time** | **Type** | **QR code** |
| --- | --- | --- | --- | --- | --- | --- |
| 1 | Neck stretching | Standing  &  Chair | At least 1/  Back and forth, side to side | 5 s | Stretching | 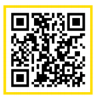 |
| 2 | Shoulder  rotation  stretching |  | At least 5/  Back and forth | 5 reps | Stretching |  |
| 3 | Row or  Shoulder  retraction |  | At least 5/  Back and forth | 5 s | Stretching |  |
| 4 | Clasp with  a small ball |  | At least 5 /  Right hand and left hand | 5 s | Resistance,  Isometric |  |
| 5 | Pec-dec fly |  | At least 5 | 5 s | Resistance,  Isometric |  |
| 6 | Pendulum  exercise |  | At least 10/  surgery arm (only) | 5 rep | Stretching |  |
| 7 | Child pose |  | At least 1 | 5 s | Stretching |  |
| 8 | Back extension  exercises |  | At least 1 | 5 s | Stretching |  |
| 9 | Pelvic tilt  on wall |  | At least 5 | 5 s | Resistance,  Isometric |  |
| 10 | Calf raise  exercise |  | At least 5 | 3 s | Resistance |  |
| 11 | Seated leg extension on chair |  | At least 5 | 5 s | Resistance |  |

**Supplementary 3.** Daily exercise program of the study (low intensity; *Phase 1*)

| **No** | **Content** | **Psition** | **Frequency** | **Time** | **Type** | **QR**  **code** |
| --- | --- | --- | --- | --- | --- | --- |
| 1 | Neck stretching | Standing  &  Chair | At least 1 /  Back and forth, side to side | 5 s | Stretching | 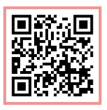 |
| 2 | Shoulder rotation  stretching |  | At least 5 /  Back and forth | 5 reps | Stretching |  |
| 3 | Row or  Shoulder  retraction |  | At least 5 /  Back and forth | 5 s | Stretching |  |
| 4 | Clasp with  a small ball |  | At least 5 /  Right hand and left hand | 5 s | Resistance,  Isometric |  |
| 5 | Pec-dec fly |  | At least 5 | 5 s | Resistance, |  |
| 6 | Pendulum exercise |  | At least 5/  surgery arm(only) | 5 reps | Isometric Stretching |  |
| 7 | Child pose |  | At least 1 | 5 s | Stretching |  |
| 8 | Back extension  exercises |  | At least 1 | 5 s | Stretching |  |
| 9 | Pelvic tilt on wall |  | At least 5 | 5 s | Resistance,  Isometric |  |
| 10 | Seated leg  extension on chair |  | At least 5 | 5 s | Resistance |  |
| 11 | Calf raise  exercise |  | At least 5 | 5 s | Resistance |  |

**Supplementary 4.** Daily exercise program of the study (low-to-moderate intensity; *Phase 2*)

| **No** | **Content** | **Psition** | **Frequency** | **Time** | **Type** | **QR**  **code** |
| --- | --- | --- | --- | --- | --- | --- |
| 1 | Neck stretching | Standing  &  Chair | At least 1 /  Back and forth, side to side | 10 s | Stretching | 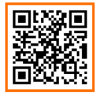 |
| 2 | Shoulder rotation  stretching |  | At least 5 /  Back and forth | 5 reps | Stretching |  |
| 3 | Row or  Shoulder  retraction |  | At least 5 /  Back and forth | 5 s | Stretching |  |
| 4 | Clasp with  a small ball |  | At least 5 /  Right hand and left hand | 5 s | Resistance,  Isometric |  |
| 5 | Pec-dec fly |  | At least 5 | 5 s | Resistance, |  |
| 6 | Pendulum exercise |  | At least 5/  surgery arm(only) | 5 reps | Isometric Stretching |  |
| 7 | Child pose |  | At least 1 | 5 s | Stretching |  |
| 8 | Back extension  exercises |  | At least 1 | 5 s | Stretching |  |
| 9 | Pelvic tilt on wall |  | At least 5 | 5 s | Resistance,  Isometric |  |
| 10 | Seated leg  extension on chair |  | At least 5 | 5 s | Resistance |  |
| 11 | Calf raise  exercise |  | At least 5 | 5 s | Resistance |  |

**Supplementary 5.** Daily exercise program of the study (moderate-intensity; *Phase 2*)

| **No** | **Content** | **Psition** | **Frequency** | **Time** | **Type** | **QR**  **code** |
| --- | --- | --- | --- | --- | --- | --- |
| 1 | Neck stretching | Standing  &  Chair | At least 1  Back and forth, side to side | 5 s | Stretching | 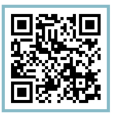 |
| 2 | Shoulder rotation |  | At least 5 /  Flexion and Abduction | 5 reps | Stretching |  |
| 3 | Cross chest stretching |  | At least 5 | 5 s | Stretching |  |
| 4 | Scapular retraction |  | At least 5 | 10 s | Stretching |  |
| 5 | Bird Dog |  | At least 5 | 10 reps | Resistance,  Isometric |  |
| 6 | Child pose |  | At least 1 | 10 s | Stretching |  |
| 7 | Back extension  exercises |  | At least 1 | 10 s | Stretching |  |
| 8 | Pelvic tilt on wall |  | At least 10 | 5 s | Resistance,  Isometric |  |
| 9 | chest stretching |  | At least 10 | 10 s | Stretching |  |
| 10 | Chair squat |  | At least 3 | 10 reps | Resistance,  Isometric |  |

**Supplementary 6.** Daily exercise program of the study (moderate-to-vigorous intensity; *Phase 3*)

| **No** | **Content** | **Psition** | **Frequency** | **Time** | **Type** | **QR**  **code** |
| --- | --- | --- | --- | --- | --- | --- |
| 1 | Neck stretching | Standing  &  Chair | At least 1  Back and forth, side to side | 5 s | Stretching | 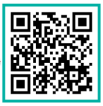 |
| 2 | Modified  chest stretching |  | At least 1 | 10 reps | Stretching |  |
| 3 | Cross chest stretching |  | At least 10 | 5 s | Stretching |  |
| 4 | Scapular retraction |  | At least 10 | 5 s | Stretching |  |
| 5 | Chair squat |  | At least 1 | 10 reps | Resistance,  Isometric |  |
| 6 | Child pose |  | At least 1 | 10 s | Stretching |  |
| 7 | Back extension  exercises |  | At least 2 | 5 s | Stretching |  |
| 8 | Bird Dog |  | At least 10 | 5 reps | Resistance,  Isometric |  |
| 9 | Pelvic tilt on wall |  | At least 10 | 5 s | Resistance,  Isometric |  |
| 10 | chest stretching |  | At least 1 | 10 s | Stretching |  |
| 11 | Y stretching |  | At least 1 | 10 s | Stretching |  |

**Supplementary 7.** Daily exercise program of the study (vigorous intensity; *Phase 3*)

| **No** | **Content** | **Psition** | **Frequency** | **Time** | **Type** | **QR**  **code** |
| --- | --- | --- | --- | --- | --- | --- |
| 1 | Neck stretching | Standing  &  Chair | At least 1  Back and forth, side to side | 5 s | Stretching | 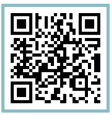 |
| 2 | Modified  chest stretching |  | At least 1 | 10 reps | Stretching |  |
| 3 | Modified  pec-dec fly |  | At least 5 | 5 s | Resistance,  Isometric |  |
| 4 | Cross chest stretching |  | At least 10 | 5 s | Stretching |  |
| 5 | Bicycle crunch |  | At least 1 | 10 reps | Strength |  |
| 6 | Child pose |  | At least 1 | 10 s | Stretching |  |
| 7 | Back extension  exercises |  | At least 2 | 5 s | Stretching |  |
| 8 | Bird Dog |  | At least 10 | 5 reps | Resistance,  Isometric |  |
| 9 | Wall push-up |  | At least 1 | 10 reps | Resistance,  Isometric |  |
| 10 | chest stretching |  | At least 1 | 10 s | Stretching |  |
| 11 | Y stretching |  | At least 1 | 10 s | Stretching |  |
| 12 | Dynamic squat |  | At least 1  Back and forth, side to side | 10 reps | Resistance,  Isometric |  |
